# The correlation among residual nitrites, biogenic amines, N‐nitrosamine formation, and degradation occurrence of punicalagin α/β, rosmarinic acid, carnosol, and carnosic acid in extract‐treated sausage during storage

**DOI:** 10.1002/fsn3.3498

**Published:** 2023-06-15

**Authors:** Elahe Abedi, Atefeh Tavakoli, Simin Zamanizadeh, Shahrzad Maleki, Amir Reza Jassbi

**Affiliations:** ^1^ Department of Food Science and Technology, Faculty of Agriculture Fasa University Fasa Iran; ^2^ Medicinal and Natural Products Chemistry Research Center Shiraz University of Medical Sciences Shiraz Iran; ^3^ Department of Civil Engineering, Faculty of Engineering Fasa University Fasa Iran

**Keywords:** biogenic amines, degradation kinetic, *Punica granatum*, residual nitrites, *Salvia eremophila*

## Abstract

The aim of this study was to investigate the relation between residual α‐ and β‐punicalagin in *Punica granatum* L.; PPE and rosmarinic acid, carnosol, and carnosic acid in *Salvia eremophila* (SE) with residual nitrites, biogenic amines (cadaverine, putrescine, and histamine), N‐nitrosodimethylamine (NDMA), microbial counts, lipid oxidation indices, and color values in extract‐treated sausage over 14 days of storage. Sausage containing SE + nitrite 60 ppm (SSN) showed minimum levels of the residual nitrites (13.14 mg/kg), NDMA (0.74 ± 0.05 μg/kg), and biogenic amine (histamine, 1.8 mg/kg; cadaverine, 3.7 mg/kg; and putrescine, 4.3 mg/kg) due to retarded degradation rate of 285.84–216.44 mg/kg; rosmarinic acid, 41.62–33.16 mg/kg; carnosol, and 88.70–76.73 mg/kg; carnosic acid over storage time. The first‐order kinetic model fitted well for the degradation of rosmarinic acid and carnosol acid in SSN sample. TBA value remained below the threshold limit (0.32 mg kg^−1^) through 14 days for SSN. Second‐order and zero‐order reaction models had the best agreement with sausages' PV and TBA values, respectively. After 2 weeks of storage, *E. coli* and *Cl. perfringens* counts in the SN120 (sausage containing 120 ppm nitrite) and SSN were significantly lower than the other samples (*p* < .05), with the values 2.1 and 1.5 log cfu/g for SN120 and 2.2 and 1.6 log cfu/g for SSN formulation. Conversely, oxidation indices, residual nitrites, NDMA, and biogenic amine increased in sausage samples containing PPE extracts (SPN) owing to total degradation of α‐ and β‐punicalagin during storage. The results indicated that SE can be used as potential co‐preservative by reducing the levels of required nitrite in food industry.

## Graphical abstract

The use of natural antioxidants in meat products has been extensively studied. However, no reports have been found regarding the accumulation of antioxidant residues in meat products over the storage time in cold storage by LC‐DAD‐MS analysis. To manufacture of *Punica granatum* L. and *Salvia eremophila* extracts ‐treated sausage, the extracts were prepared under acidic and non‐acidic aqueous of methanol solvents. After that the optimized extracts were chosen and incorporated to reduced nitrite sausage concerning to maximum DPPH radical scavenging and anti‐microbial activity. The amount of punicalagins, rosmarinic acid, carnosol and carnosic acid of extracts were determined in the sausage formulations during different time points of cold storage. Finally, oxidative indices (PV and TBA), microbial count and color indices (L*, b*, c*, oxymyoglobin and metmyoglobin) of different sausage formulations are compared to control sausage (120 ppm).

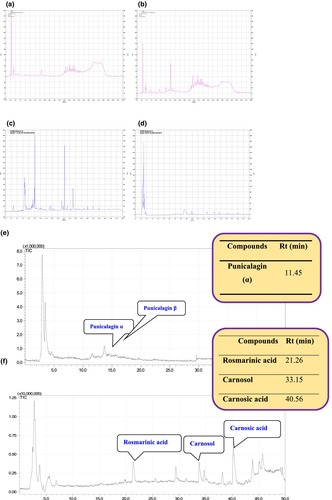

## 1 INTRODUCTION

Sausage is considered as a highly perishable meat product because it is manufactured from fresh ground meat which is desirable for spoilage and pathogenic microbial growth. Moreover, it contains a high‐fat level, facilitating lipid oxidation. Thereby, this product requires to be preserved its qualities (Alirezalu et al., 2020; Alirezalu, Hesari, Yaghoubi, et al., 2021; Hugo & Hugo, 2015). Nitrate and nitrite have been widely utilized in meat products to extend the shelf life by prohibiting lipid oxidation and microbial spoilage induced by *Clostridium perfringens*, *Escherichia coli*, *Staphylococcus aureus*, and *Salmonella enteritidis* (Peighambardoust et al., 2022; Saggiorato et al., 2012; Sepahvand et al., 2021; Smaoui et al., 2017; Tosati et al., 2017) and also improve color and flavor characteristics of the end products (Glorieux et al., 2019; Smaoui et al., 2017). Despite all of these benefits, leading concerns for nitrite utilization are related to the formation of nitrosamine derivatives. Nitrosamine compounds include N‐nitrosodiethylamine, N‐nitrosodimethylamine, N‐nitrosodibutylamine, N‐nitrosopiperidine. and N‐nitrosopyrrolidine, which can potentially increase risk of cancer, methemoglobinemia, nasopharynx, esophagus, stomach, pancreas, colorectal and brain cancer, and other tumors which remains inconclusive (Alirezalu, Movlan, Yaghoubi, et al., 2021; Alirezalu, Yaghoubi, Nemati, et al., 2021; Alirezalu, Yaghoubi, Poorsharif, et al., 2021; Deng et al., 2022; Wang et al., 2021; Yaghoubi et al., 2021).

The use of plant‐based extracts as antimicrobials and antioxidants in food products has gained great attention (Ayaseh et al., 2022; Mahajan et al., 2015; Yaghoubi et al., 2023). In this regard, replacing synthetic antioxidants with natural compounds in various products causes to postpone oxidative degradation of lipids and microbial growth and improves nutritional value of foods (Mahajan et al., 2015).

Pomegranate (*Punica granatum* L. *Punicaceae*) and Sages (*Salvia* L.) are native medicinal plants from Iran, being characterized by enormous antioxidant, antimicrobial, cytotoxic, phytotoxic, antiprotozoal, insecticide, antileishmanial, and antimalarial effects (Aloqbi et al., 2016; Asadollahi et al., 2019; Feng et al., 2017; Ismail et al., 2012; Jassbi et al., 2016).

Moreover, numerous studies stated that pomegranate and sages as natural antioxidants have been used to prohibit lipid oxidation and discoloration and extend shelf life of meat products (Kumar et al., 2015; Shah et al., 2014). Furthermore, natural antioxidants act as the nitrite scavenging and had an important role to inhibit N‐nitrosamine formation (Yao et al., 2016).

The use of natural antioxidants in meat products has been extensively studied. Therefore, the objective of this study was to assess the effect of using *Punica granatum L*. and *Salvia eremophila* extracts with and without the use of nitrite in sausage formulation on the residual nitrites, biogenic amines (cadaverine, putrescine, and histamine), N‐nitrosamine, oxidative indices (PV and TBA), microbial count, and color values (L*, b*, c*, oxymyoglobin and metmyoglobin). In addition, the residual amount of punicalagins, rosmarinic acid, carnosol, and carnosic acid were determined in the sausages formulated with extracts during storage time.

## 2 MATERIALS AND METHODS

### 2.1 In vitro studies

#### 2.1.1 Plant material extraction

The peel of *P. granatum* was collected from a garden in Ghasrodasht of Shiraz (Iran, September 2019) at the fruit ripening stage. The aerial parts of the *S. eremophila* were also collected from Shiraz surrounding during the flowering stage of the plants. After grinding, the fresh pomegranate peels and aerial part of sage were subjected to solvent extraction using aqueous methanol (80% v/v) in the absence and presence of acetic acid (HOAc, 5% v/v). The PPE and SE were filtered and concentrated in vacuum oven at 40°C by following the method of Hashemi et al. (2018).

#### 2.1.2 HPLC analysis of pomegranate and sage extracts

The reversed‐phase HPLC analyses of plant extracts were conducted by a Knauer analytical HPLC with a K‐1001 pump. The separations were carried out on a Eurospher‐100 C_18_ column (Eurospher‐100 C_18_, 250 mm × 4.6 mm, Knauer, Germany). The samples were separated with linear gradient elution. The gradient method was solvent A: 0.125% H_3_PO_4_ in ultrapure water; and supplementing the solvent with acetonitrile as the solvent B as: 0–5 min, 5% of B; 5–10 min, 5%–10% of B; 10–20 min, 10% of B; 20–40 min, 10%–40% of B; 40–45 min, 40%–60% of B; 45–50 min, 60%–100% of B; 50–55 min, 100% of B, and 55–60 min, 5% of B. In the following gradient program, 0.25% H_3_PO_4_ is dissolved in ultrapure water (solvent A), with increasing solvent strength with acetonitrile as solvent B as: 0–6 min, 0%–12% of B; 6–10 min, 12%–18% of B; 10–30 min, 18%–58% of B; 30–35 min, 58%–80% of B; 35–45 min, 80% of B, and 45–50 min, 80%–0% of B. The four‐channel K‐2600 Ultraviolet detector was set at λ210, 254, 320, and 365 nm (Asadollahi et al., 2019; Amir Reza Jassbi et al., 2016).

#### 2.1.3 Total phenolic content

Folin–Ciocalteu was used to measure total phenolic compounds in the resulting extracts as described by Pakfetrat et al. (2020). In order to estimate the total content of phenolics in plant compounds, a standard curve was plotted using various concentrations of gallic acid as mg/g.

#### 2.1.4 Radical‐scavenging activity using DPPH assay

The antioxidant capacity of sage and pomegranate extracts was evaluated using the modified method conducted by Zare et al. (2021). The percentage of inhibition was calculated from the following Equation (1):

(1) $$\text{DPPH radical scavenging activity}\;\left(\%\right)=\left[\left(A_{\text{Control}}–A_{\text{Sample}}\right)/A_{\text{Control}}\right]\times 100$$

*A*_sample_ represents the absorbance of DPPH radical solution mixed with sample extract, while *A*_Control_ represents the absorbance of DPPH radical solution in methanol. Quercetin was used as standards. Based on linear regression equations relating to levels of extract versus DPPH inhibition percentage, the IC_50_ values were calculated using Curve Expert and Microsoft Excel.

#### 2.1.5 Antibacterial minimum inhibitory concentration

*Escherichia coli* strain (ATCC 25922) as gram‐negative and *Clostridium perfringens* strain (RITCC 2752) as gram‐positive bacteria were purchased from the Iranian Research Organization for Science and Technology. Stock cultures including Mueller–Hinton Broth (MHB; Merck, Darmstadt, Germany) for *E. coli* and Fluid Thioglycollate Media (FTG; Gibco, Paisley, Scotland) for *C. perfringens* were prepared and kept at 4°C until further use (Moarefian et al., 2012). Antimicrobial effect of sage and pomegranate extracts was evaluated by MIC (minimum inhibitory concentration) determinations. In order to determine MIC, a broth dilution method was used with some modifications (Moarefian et al., 2012). *E. coli* suspension was obtained by microbial count of 1.5 × 10^8^ cfu/mL and turbidity equivalent to 0.5 McFarland standard; 100 mL of SE and PPE dispersion in Tween 20 was mixed to 100‐mL MHB medium in microtiter plate wells and inoculated with 10‐mL *E. coli* suspension at 37°C for 24 h. Bacterial growth was evaluated by turbidity developed in broth culture and for determining MIC, cell suspensions were subcultured on MacConkey medium (Merck, Darmstadt, Germany) in triplicate. The same method was applied to measure MIC concentrations of the SE and PPE against *C. perfringens*. Bacterial suspension was prepared in FTG with microbial count of 10^8^ cfu/mL. SE and PPE dilution was done in 1 mL of FTG medium in test tube, and 100 mL of *C. perfringens* was inoculated. Liquid paraffin (0.5 mL) was added on top of the culture medium to maintain anaerobic conditions in test tubes and incubation was done at 37°C for 24 h. Turbidity was used as indicator of growth to determine MIC content.

### 2.2 In situ studies

#### 2.2.1 Sausage production

Sausage formulations were produced from ground beef. Three sources of ground beef from chuck section were mixed together, thereafter divided into three groups and three sausage formulations were prepared. Sausage samples were manufactured by using the following formula: 80% beef meat, 1% soybean powder, 2% gluten, 4% wheat flour, 0.4% Na_5_P_3_O_10_, and 0.04% ascorbic acid.

Brines were formulated by salt (9%‐degree baume), NaNO_2_ (120 or 60 mg/kg meat), and extracts (1% SE and PPE). The paste was divided into different batches and the brines with or without nitrites and extracts were added into each group according to the paste weight, after that, mixtures were blended in a cutter (Seydelmann, Germany). (1) 120 ppm nitrite‐containing sausage (SN120), (2) 60 ppm nitrite‐containing sausage (SN60), (3) sausage containing 1% SE (SS), (4) sausage containing 1% PPE (SP), (5) sausage containing 1% PPE and nitrite 60 ppm (SPN), (6) sausage containing 1% SE and nitrite 60 ppm (SSN). The batches were filled in polyamide casings separately. Cured sausages were heat‐dried for 1 h at 50°C and stored for 14 days for ripening.

#### 2.2.2 Sausage preparation for HPLC and LC‐DAD‐ESIMS analysis

First, weighing (5 g) of the samples and rinsing with n‐hexane (15 mL) were executed, followed by their transfer to a separatory funnel having 5‐mL n‐hexane and then extraction using n‐hexane‐saturated acetonitrile (150 mL). The organic solvent layer was moved to a different funnel for the residual removal. A round‐bottom flask was employed for the collection of acetonitrile phase and an SB‐1200 water bath (Eyela, Japan) along with an N‐1200A vacuum rotary evaporator (Eyela, Japan) was applied for the evaporation of this phase to the final 3‐ to 4‐mL volume. Small solvent portions (1:1 ratio of acetonitrile: iso‐propanol) were used for rinsing the flask, succeeded by its transfer to a 10‐mL flask and collection of the exact amount through repeating the rinsing process (Choi et al., 2019).

A Shimadzu LCMS‐2010EV equipped with an ESI mass column and SPD‐M20A diode array detector was used for the LC‐DAD‐ESI‐MS analyses. Column's Shim Pack XR‐ODS C 18 column (75 × 3.0 mm, 2.2 μm) and LC pump (LC 20 AD) with flow rate 0.25 mL/min was used. Injection volume was 5 μL of the 10 times‐diluted the sample solutions. In negative modes, the ESI was used as an ionizing source. The MS parameters include as: MS detector voltage: ±1.5 kV, interface: ±4.5 kV, CDL: ±10 V, and Q‐array (Rf: ±150 V) voltages uploaded from the tuning file. There were three parameters that were scanned: mass range 100–1000 units, nebulizer gas N_2_, flow rate 1.5 L/min, and heat block and CDL temperatures 230 and 275°C, respectively. On a DAD detector, the Ultraviolet spectrum was recorded between 190 and 600 nm, and a temperature of 40°C was set for both the cell and the column. The HPLC apparatus was a Knauer analytical HPLC with an Azura (p 6.1 L) pump and a Knauer Ultraviolet detector (2.1 L). The analytical column was a Eurospher‐100 C18 (Eurospher‐100 C18, 250 × 4.6 mm, Knauer, Germany).

In gradient elution, the mobile phase consisted of A (1% acetic acid in water) and B (methanol) as follows: 0–20 min linear from 10% to 65% A; 20–40 min linear from 65% to 100% A; 40–45 min, 100% A; 45–47 min linear from 100% to 10% A; finally, holding for 3 min. A 0.45‐m membrane filter (Whatman, Amersham, UK) was used to filter the mobile phase and degassed under vacuum. The flow rate and injection volume were set at 1.0 mL/min and 20 μL.

#### 2.2.3 Determination of peroxide value (PV) and thiobarbituric acid (TBA) value

PVs (primary lipid oxidation indices) and TBAs (secondary lipid oxidation indices) were determined by the method of Naveena et al. (2013).

#### 2.2.4 Color evaluation

The color of samples was measured after 1, 7, and 14 days using the Hunter‐Lab ColorFlex Colorimeter (Hunter Associated Lab, Inc., Reston, Virginia, USA). The amount of oxymyoglobin (OxyMb) and metmyoglobin (MetMb) in sausages was evaluated according to Ghaderi‐Ghahfarokhi et al. (2016). The OxyMb and MetMb contents were calculated using Equations 2 and 3:

(2) $$\text{OxyMb}\;\left(\%\right)=\left(0.88{\text{2R}}_{1}–1.26{\text{7R}}_{2}+0.80{\text{9R}}_{3}–0.361\right)\times 100$$

(3) $$\text{MetMb}\;\left(\%\right)=\left(-2.54{\text{1R}}_{1}+0.77{\text{7R}}_{2}+0.80{\text{0R}}_{3}+1.098\right)\times 100$$

where R1, R2, and R3 are the absorbance ratios of A_572_/A_525_, A_565_/A_525_, and A_545_/A_525_, respectively.

#### 2.2.5 Residual nitrite determination

Nitrite level was identified concerning to the previous report of Wang et al. (2021). The dry sausage (5 g) was minced by meat grinder and placed into conical flask, saturated borax solution (12.5 mL), and then distilled water (150 mL; 70°C) was added. The resulting mixture was placed in boiling water bath (15 min), then cooled to room temperature. Reagent K_4_Fe(CN)_6_·3H_2_O (5 mL; 106 g/L) was added to the mixture and shaken. Afterward, Zn (CH_3_COO)_2_·2H_2_O solution (5 mL; 220 g/L) was added. Subsequently, distilled water was incorporated into the mixture to reach the final volume of 200 mL. Using filter paper (20 μm pore size, Whatman, Mosu Scientific Equipment Co., China), the mixture was filtered and collected. *p*‐aminobenzenesulfonic acid (2 μL; 4 g/L) was added to the sausage filtrate (40 mL). After standing for 5 min, N‐ethylenediamine standard solution (1 mL; 2 g/L) was inserted into the mixture and stood at room temperature for 15 min. Absorbance of unknown sample and standard nitrate were measured at 538 nm by spectrophotometer (AA‐680, Shimadzu, Kyoto, Japan).

#### 2.2.6 Determination of biogenic amines

Biogenic amine content was measured according to Wang et al. (2021). Standard solutions (0, 2.5, 5, 10, 20, and 40 mg/mL) of cadaverine, putrescine, and histamine (Sigma‐USA) were made using perchloric acid (0.4 M) and stored at 4°C. The sample (5 g) was homogenized in perchloric acid (20 mL; 0.4 M) for 4 min using homogenizer (Ultra‐Turrax T50, IKA‐Werke, Staufen, Germany) followed by centrifugation at 2500 × *g* (4°C; 10 min) (Thermo Fisher company, USA). The supernatant was collected with 50‐mL perchloric acid (0.4 M). Aqueous extract of sausage (1 mL) was mixed with sodium hydroxide (200 μL; 2 M) to produce the alkaline solution. The saturated sodium bicarbonate solution (300 μL) was added for buffering, followed by dansyl chloride (2 mL; 10 mg/mL) was combined to react at 40°C for 30 min in the dark, and the reaction stopped using 100‐μL ammonia. Finally, the resulting mixture was adjusted with acetonitrile to volume 5 mL and centrifuged at 2500 × *g* (4°C for 3 min). The biogenic amines of final mixture were analyzed using high‐performance liquid chromatography with C18 column (ID 5 μm, 4.6 mm × 150 mm, Agilent, USA) at 254 nm. The elution was carried out using acetonitrile (solvent A) and water (solvent B). The gradient elution was conducted as follows, 0–5 min, 65% A; 5–20 min, 70% A; 20–25 min, 100%.

#### 2.2.7 N‐nitrosamines analysis

Ten grams of minced sausages was mixed with NaOH solutions (10 mL; 0.1 mol/L) and sonicated for 15 min at 25°C. Then, methanol (20 mL) was added and homogenized for 3 min. After centrifugation at 10,000 *g* for 10 min at 4°C, homogenates were filtered with filter paper. The methanol–water extract (15 mL) was combined with NaCl solution (5 mL; 20%) and loaded on a ChemElut column with a capacity of 20 mL. A 50‐mL solution of dichloromethane was used to elute the column after it had been equilibrated for 20 min. After being concentrated to 1 mL under reduced pressure and evaporated in a water bath under nitrogen conditions (40°C), the eluent was analyzed by GC coupled to a quadrupole mass selective spectrometer (Agilent Technologies, Palo Alto, CA, USA). Agilent DB‐5MS column (30 m × 0.25 mm × 0.25 μm) with injector temperature 230°C was used for chromatographic separation. Specifically, the oven was programmed to run for 2 min at 50°C, 2 min at 150°C, 5 min at 250°C, 1 min at 20°C/min, and 1 min at 280°C. There was a velocity of 1.0 mL/min for the helium carrier gas. Using electron‐impact ionization, ions were produced. There were temperatures of 230°C and 250°C for the ion source and transfer line. The injection volume was 1 μL. By comparing retention indices and MS spectra with pure standards, the compounds were identified.

#### 2.2.8 Microbial analysis

A portion (10 g) of sausages aseptically transferred into individual stomacher bag containing 90 mL of Ringer's solution. Samples were homogenized using stomacher for 2 min to prepare the initial dilution. *E. coli* was counted following 48‐h incubation at 37°C on Baird Parker agar supplemented with sorbitol McConkey agar (Difco) (Ghaderi‐Ghahfarokhi et al., 2016; Ibrahim et al., 2011). The stock culture of the *Cl. Perfringens* strain was prepared in Duncan and Strong medium. Spore suspension was moved to microtubes and centrifugated. The spores were resuspended in sterile peptone water (0.1%), heat shocked for 20 min at 75°C, and manually blended with meat paste. The paste was cooked in water bath for 1 h at 75°C and then cooled at 20°C. For enumeration of *Cl. Perfringens*, sample (10 g) was homogenized in sterile salt solution (90 mL, NaCl, 0.85%) using a sterile stomacher (Sanyo, Japan) and kept in anaerobic culture in sulfite polymyxin sulfadiazine agar.

### 2.3 Kinetic modeling

Zero‐order, first‐order, and second‐order kinetic models were used to determine the reaction rates of various parameters including rosmarinic acid, carnosol, and carnosic acid, as well as oxidative indices (PV and TBA). For the zero‐order reaction, the equation can be expressed as follows:

(4) $$\frac{\mathrm{dC}}{\mathrm{dt}}=-k$$

(5) $$C=C_{0}-\mathrm{kt}$$

where *C*_*0*_ is the initial concentration, *C* is the concentration at time *t*, and *k* is the reaction rate constant of the zero‐order reaction.

Assuming first‐order kinetics, the equation can be expressed as follows (Zhang et al., 2012):

(6) $$\frac{\mathrm{dC}}{\mathrm{dt}}=-\mathrm{kC}$$

(7) $$\ln\frac{C}{C_{0}}=-\mathrm{kt}$$

(8) $$C=C_{0}e^{-\mathrm{kt}}$$

where *k* is the reaction rate constant of the first‐order reaction.

For the second‐order kinetics, the rate of reaction can be determined as follows:

(9) $$\frac{\mathrm{dC}}{\mathrm{dt}}=-{\mathrm{kC}}^{2}$$

(10) $$\frac{1}{C}=\frac{1}{C_{0}}+\mathrm{kt}$$

(11) $$C=\frac{C_{0}}{1+C_{0}\mathrm{kt}}$$

where *k* is the reaction rate constant of the second‐order reaction.

### 2.4 Statistical analysis

The statistical analyses were carried out using IBM SPSS statistics, version 22.0 (IBM Corp, Armonk, NY, USA). Statistical assumptions such as normality of data distribution and homogeneity of variances were tested by Shapiro–Wilk and Levene's tests, respectively, before conducting one‐way ANOVA to check for differences regarding the effect of different extracts (PPE and SE) and the effect of different storage time on the oxidation indexed, biogenic amines, residual nitrites, NDMA, color, and microbial counts. Duncan's multiple range test was used for post hoc analyses. Statistical significance was set at 5%. The results were presented as mean ± SD.

## 3 RESULTS AND DISCUSSION

### 3.1 Phenolic compounds, radical scavenging, and antimicrobial activities of PPE and SE

**FIGURE 1:**
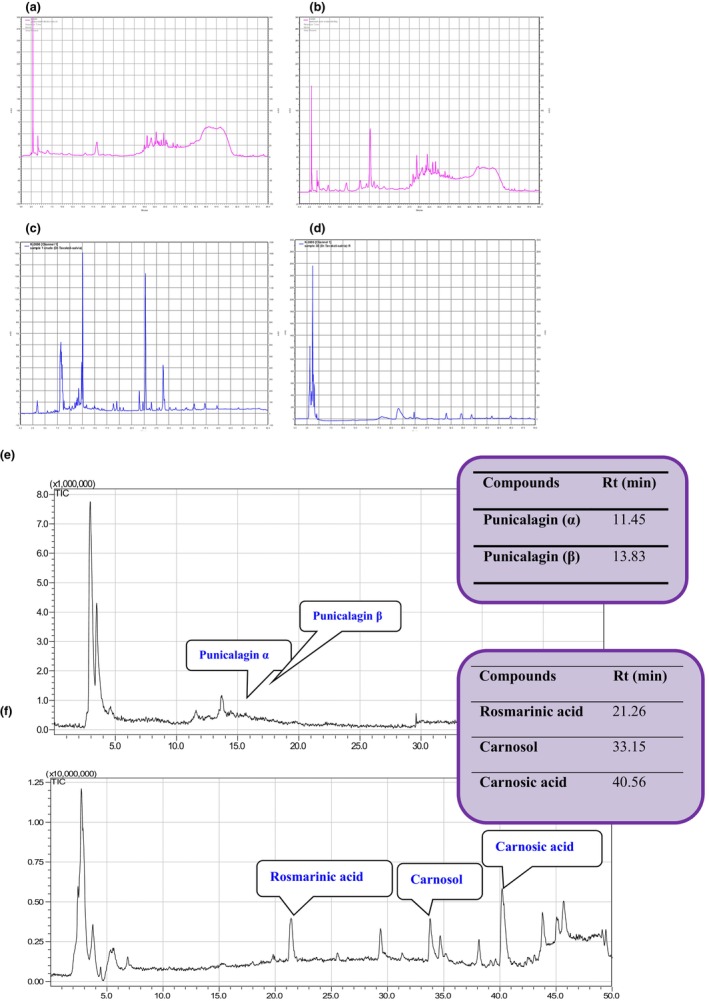
HPLC analysis of pomegranate and salvia; pomegranate crude extract (a), pomegranate crude extract + acetic acid (b), salvia crude extract (c), salvia crude extract + acetic acid (d), LC‐TCI of the pomegranate extract (e), and salvia (f) before addition to the sausage.

The level of phenolic compounds (145.43 ± 3.82 mg gallic acid/g plant) and (123.37 ± 2.83 mg gallic acid/g plant) which was in accordance with lower DPPH IC_50_ (19.28 ± 1.35 μg/mL) and (27.09 ± 1.65 μg/mL) in acidic and nonacidic crud alcoholic of pomegranates resulting fractions, respectively (Table 1 and Figure 1). Pomegranate peel extract was mostly discovered as a wealthy source of antioxidants because hydrolyzable tannins such as punicalagin and ellagitannins are predominantly located in the fruit mesocarp and peel of pomegranates which provide 92% of antioxidant activity (Aloqbi et al., 2016; Feng et al., 2017). The radical scavenging activity of extracts in acid was markedly (*p* < .05) more than nonacid extraction. Acidifying the extraction solution affects the dissociation of phenols and acid‐induced dissociation, the gallotannins, ellagitannins, gallagic acid, and gallagyl esters are hydrolyzed to several ions and numerous compounds such as gallic acid, ellagic acid, quercetin, punicalagin, etc. As a result, the phenolic contents and antioxidant potential of the PPE extracts increased (Table 1). Obtained IC_50_ in the present study was highly greater than those previously reported for pomegranate peel extract in a DPPH test (IC50 4.9 μg/mL) (Hugo & Hugo, 2015). In previous literatures, aqueous pomegranate peel extract exhibited high phenolic level as 161.25 and 140 mg/g in terms of catechin equivalent (Hugo & Hugo, 2015).

**TABLE 1:** DPPH radical scavenging activity (%), IC_50_ (μg/mL), total phenol (mg gallic acid/g plant), and MIC (*Escherichia coli* and *Clostridium perfringens*) by pomegranate and salvia extract (1%).

	Quercetin	Pomegranate		Salvia	
		Methanol extraction + acetic acid	Methanol extraction	Methanol extraction + acetic acid	Methanol extraction
IC_50_ (μg/mL)	20.18 ± 1.82D	19.28 ± 1.35C	27.09 ± 1.65B	59.76 ± 2.51A	62.26 ± 4.89A
Total phenol (mg gallic acid/g plant)	—	145.43 ± 3.82A	123.37 ± 2.92B	79.04 ± 4.26C	77.67 ± 4.83C
MIC (mg/L *Escherichia coli)*	—	3.5 ± 0.1B	3.5 ± 0.1B	0.85 ± 0.2A	0.85 ± 0.1A
MIC (mg/L *Clostridium perfringens)*	—	2.5 ± 0.1B	2.5 ± 0.2B	0.35 ± 0.1A	0.35 ± 0.1A

*Note*: Comparison procedure according to SAS test was carried out for the data shown in each raw. Values are the average of triplicates ± standard deviation. Different capital letters in each row indicate significant statistical difference (*p* ≤ .05).

SE showed DPPH free radical scavenging activity with an IC_50_ (59.76 ± 2.51 μg/mL and 62.26 ± 4.89 μg/mL) with total phenolic content (79.08 ± 4.26 mg gallic acid/g plant and 77.67 ± 4.83 mg gallic acid/g plant) under acidic and nonacidic conditions, respectively (Table 1). No significant difference was observed between acidic and nonacidic treatments. Significantly (*p* ˂ .05) lower antioxidant activity was demonstrated for SE compared to quercetin (20.18 μg/mL) and pomegranate. Three major compounds in *S. eremophila*, namely, rosmarinic acid, carnosol, and carnosic acid were identified (Figure 1). Rosmarinic acid is characterized as the major hydrophilic compound while carnosol and carnosic acid are main lipophilic constituents. Rosmarinic acid as phenylpropanoid compound comprises an ester of caffeic acid and 3,4‐dihydrophenyl lactic acid. Carnosic acid is a labdane‐type diterpene and carnosol is a major oxidation product of carnosic acid which are proven with high antioxidative capacities (Birtić et al., 2015). Concerning data obtained by Table 1, from the antioxidative point of view, the pomegranate is depicted higher antioxidant activity.

Bioactive compounds derived from pomegranate and sage have been represented to be a good alternative to prevent growth of various pathogenic bacteria. The antimicrobial activity of PPE and SE against common food spoilage and pathogenic bacteria such as *E. coli* and *Cl. perfringens* was examined (Table 1). In the case of gram‐negative bacteria (*E. coli*), there was no significant difference (*p* > .05) in MIC of pomegranate and sage extracts under acidic and nonacidic conditions while in the case of gram‐positive bacteria (*Cl. perfringens*), the antibacterial effects of the SE extract (0.35 mg/mL media) are higher than PPE extracts (2.5 mg/mL media; Table 1). Kanatt et al. (2010) stated that pomegranate peel extract at concentration of 0.01% could sufficiently prevent gram‐positive bacteria such as Staphylococcus aureus and *Bacillus cereus*; however, concentration of 1% could not inhibit gram‐negative bacteria growth, namely, *E. coli*, *Salmonella enteritidis*, and *Pseudomonas* spp. Hugo and Hugo (2015) revealed that pomegranate extract was less effective against *E. coli* and *P. aeruginosa*.

### 3.2 LC–MS of sausage containing PPE and SE

HPLC analysis showed that the concentration of methanol extracted α‐ and β‐punicalagin was 67 and 232 mg/kg, respectively, before being added to the sausage (Figure 1). As evidenced by the LC–MS analysis, there were no punicalagins in the sausage prepared with pomegranate extract from the first to the 14th day of storage time, implying all of them had been converted or decomposed. Furthermore, the new spectra concerning the derivatives of quinic acid and the derivatives of gallic acid and sucrose are found as major compounds in pomegranate peel extract‐treated sausage (Figure S1).

The concentration of rosmarinic acid, carnosol, and carnosic acid compounds in sage extracted with methanol was 313.13, 41.61, and 86.94 mg/kg, before adding to sausage formulation which reduced to 258.06, 36.08, and 82.46 mg/kg (Table 2), respectively, in SS sausage during 14‐day storage time. The same reduction pattern was observed by rosmarinic acid (216.44 mg/kg), carnosol (33.15 mg/kg), and carnosic acid (76.73 mg/kg) in SSN sausage further 14th day of storage time (Figure S1).

**TABLE 2:** Concentration (mg/kg) and kinetic parameters for rosmarinic acid, carnosol, and carnosic acid changes in sausage samples after first, 7th, and 14th day of storage.

Sausage samples	Storage time	Rosmarinic acid	Carnosol	Carnosic acid
Sausage (1% Sage extract)	First	313.13 ± 9.15A	41.61 ± 7.07A	86.94 ± 2.19A
	7	290.31 ± 7.18B	37.31 ± 1.74C	84.71 ± 2.23B
	14	258.06 ± 12.97D	36.08 ± 1.42CD	82.46 ± 0.43C
Sausage (60 ppm NO_2_+ 1% Salvia)	First	297.53 ± 11.69B	40.62 ± 0.87B	81.78 ± 4.37C
	7	277.68 ± 2.33C	34.89 ± 1.27DE	79.9 ± 1.52D
	14	216.44 ± 6.15E	33.15 ± 0.39E	76.73 ± 3.71E

Sausage sample	Compound	First‐order kinetic		Second‐order kinetic	
		*k* (d^−1^)	*R* ^2^	*k* (kg mg^−1^ d^−1^)	*R* ^2^
Sausage (1% Sage extract) Sausage (1% Sage extract + 60 ppm NO_2_)	Rosmarinic acid	0.0159	.992	0.00005	.992
		0.0273	.945	0.0001	.941
Sausage (1% Sage extract) Sausage (1% Sage extract + 60 ppm NO_2_)	Carnosol	0.0320	.312	0.0007	.396
		0.0388	.428	0.0009	.531
Sausage (1% Sage extract) Sausage (1% Sage extract + 60 ppm NO_2_)	Carnosic acid	0.0095	.845	0.0001	.860
		0.0120	.986	0.0001	.991

*Note*: Comparison procedure according to SAS test was carried out for the data shown in each column. Values are the average of triplicates ± standard deviation. Different capital letters in each column indicate significant statistical difference (*p* ≤ .05). Same letters in each column and row indicate no significant statistical difference (*p* ≥ .05).

In SS sausage formulation, rosmarinic acid concentrations declined during storage while the ratio of carnosic acid to carnosol is not significantly affected in SS‐treated sausages (Table 2). Notwithstanding, the addition of nitrite to the SS‐treated sausages (SSN) formulation appears to accelerate the decaying of the three antioxidative substances rosmarinic acid, carnosol, and carnosic acid. These results implied that these compounds might have the ability to interact with sausage ingredients including probably nitrite to prevent its conversion to nitrosamine.

#### 3.2.1 Degradation modeling of rosmarinic acid, carnosol, and carnosic acid in the sausage samples

**FIGURE 2:**
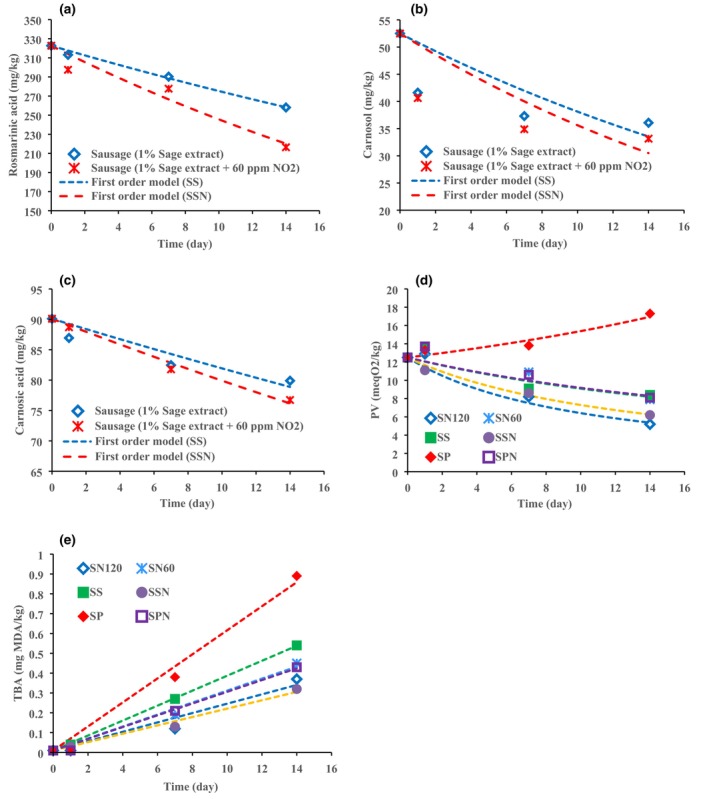
Changes in the (a) rosmarinic acid, (b) carnosol, (c) carnosic acid, changes in the PV (d), and TBA (e).

Experimental results of changes in rosmarinic acid, carnosol, and carnosic acid over time are shown in Figure 2. According to Figure 2a–c, the amounts of rosmarinic acid and carnosic acid in both SS and SSN sausage samples decrease during the storage time. Figure S1d,e exhibits that the amount of carnosol in both sausage samples rapidly decreased on the first day, after that, decreasing pattern proceeds at a slower rate and the changes are very small, so that its concentration is almost constant on day 14 compared to day 7.

In order to fit the data to the first‐ and second‐order reaction models, plots of ln (*C*/*C*_*0*_) and 1/*C* versus *t* were drawn, and *R* ^*2*^ and *k* values were calculated (Table 2). The comparison of *R* ^*2*^ values for rosmarinic acid and carnosol acid shows that both the first‐ and second‐order reaction models are in good agreement with the experimental data, while the carnosol values in both sausage samples do not fit to any of the first‐ and second‐order models. The results of fitting the data to the first‐order kinetic equation are also shown in Figure 2. The first‐order reaction rate constants (*k*) for rosmarinic acid and carnosol acid in SSN sample are greater than SS sample, which indicates that these two compounds degrade faster in SSN sample.

### 3.3 Lipid oxidation

Lipid oxidation causes sensory disorders including loss of nutrients, off‐flavor, rancid odor, drip losses, discoloration, shelf life shortening of meat product, and the accumulation of carcinogenic and/or mutagenic compounds (Aliakbarlu & Khalili Sadaghiani, 2015; Mancini et al., 2015). The oxidative quality of the products was evaluated by the determination of PV and TBA (Table 3) which are known indicators to demonstrate the amount of the primary and secondary lipid oxidation products. Table 3 depicts the PV and TBA values of sausage formulations. PV in SP samples increased from 13.3 ± 0.4 to 17.3 ± 1.3 meqO_2_/kg, whereas SPN sausage showed decreasing pattern from 13.7 ± 0.4 to 8.1 ± 0.2 on first day and 14th day, respectively. As depicted in Table 3, the PV level of SSN (6.2 ± 0.4) was significantly lower than SS (8.4 ± 0.8) and SN60 (7.9 ± 0.7) after 14‐day storage time. It is notable that acceptable threshold for rancidity perception of PVs and TBA value by consumers should be below 25 meq O_2_/kg oil and 0.5 mg MDA/kg meat, respectively (Ghaderi‐Ghahfarokhi et al., 2016). Although significant difference (*p* ˂ 0.05) was shown between PV of SN120 (5.2 ± 0.5) and SSN (6.2 ± 0.4), both PVs are acceptable for sausage formulations (˂25 meq O_2_/kg oil), indicating that reducing nitrite around 50% and using SE could progressively postpone the lipid oxidation.

**TABLE 3:** Peroxide values (meqO_2_/Kg), TBA (mg MDA/Kg), and kinetic parameters for PV and TBA of the sausage treated with pomegranate and salvia extract after first, 7th, and 14th day of storage.

Sausage samples	PV (meqO_2_/kg)			TBA (mg MDA/kg)		
	Day					
	First	7	14	First	7	14
SN120	12.9 ± 0.9aA	8.2 ± 0.2bC	5.2 ± 0.5cE	0.01 ± 0.00cC	0.12 ± 0.00bF	0.37 ± 0.03aD
SN60	13.2 ± 0.5aA	10.9 ± 0.4bB	7.9 ± 0.7cC	0.03 ± 0.00cB	0.19 ± 0.00bD	0.45 ± 0.06aC
SS	13.7 ± 0.5aA	9.1 ± 0.6bBC	8.4 ± 0.8cB	0.04 ± 0.02cA	0.27 ± 0.00bB	0.54 ± 0.14aB
SSN	12.8 ± 1.2aA	8.6 ± 0.4bC	6.2 ± 0.4cD	0.03 ± 0.00cB	0.13 ± 0.00bE	0.32 ± 0.08aD
SP	13.3 ± 0.4bA	13.8 ± 0.4bA	17.3 ± 1.3aA	0.01 ± 0.00cC	0.38 ± 0.02bA	0.89 ± 0.17aA
SPN	13.7 ± 0.4aA	10.6 ± 0.7bB	8.1 ± 0.2cC	0.01 ± 0.00cC	0.21 ± 0.00bC	0.43 ± 0.06aC

PV's kinetic parameters	Zero‐order kinetic		First‐order kinetic		Second‐order kinetic	
	k (meq kg^−1^ d^−1^)	*R* ^2^	k (d^−1^)	*R* ^2^	k (kg meq^−1^ d^−1^)	*R* ^2^
SN120	0.5362	.970	0.0618	.984	0.0076	.970
SN60	0.3045	.916	0.0298	.912	0.0030	.900
SS	0.3252	.809	0.0313	.845	0.0030	.876
SSN	0.4752	.944	0.0510	.983	0.0057	.991
SP	0.3134	.911	0.0216	.919	0.0015	.923
SPN	0.2996	.870	0.029	.895	0.0029	.906

TBA's kinetic parameters	Zero‐order kinetic		First‐order kinetic		Second‐order kinetic	
	*k* (mg kg^−1^ d^−1^)	*R* ^2^	*k* (d^−1^)	*R* ^2^	*k* (kg mg^−1^ d^−1^)	*R* ^2^
SN120	0.0236	.948	0.2762	.955	8.1456	.827
SN60	0.0302	.989	0.3049	.833	8.5314	.180
SS	0.0377	.999	0.3264	.749	8.6307	.027
SSN	0.0211	.984	0.2747	.826	8.4109	.173
SP	0.0606	.985	0.3590	.900	8.3978	.802
SPN	0.0296	.992	0.3007	.901	8.2687	.808

*Note*: Comparison procedure according to SAS test was carried out for the data shown in each row and column. Values are the average of triplicates ± standard deviation. Different lowercase letters in each row and capital letters in each column indicate significant statistical difference (*p* ≤ .05). Same letters in each column and row indicate no significant statistical difference (*p* ≥ .05). SN120 (sausage containing nitrite 120 ppm), SN60 (sausage containing nitrite 60 ppm), SS (sausage containing SE extract 1%), SP (sausage containing PEP extract 1%), SPN (sausage containing PEP extract 1% + NO_2_ 60 ppm), and SSN (sausage containing SE extract 1% + NO_2_ 60 ppm).

Regardless of treatment, TBA increased throughout 14‐day cold storage. TBA values of SPN (0.43 ± 0.06) were significantly (*p* ˂ 0.05) lower than SP (0.89 ± 0.17) (Table 3). There is no significant difference (*p* > 0.05) in TBA values between SN120 (0.37 ± 0.03) and SSN (0.32 ± 0.08), indicating concomitantly utilization of nitrite (60 mg/kg) with sage extract can be efficient against lipid oxidation via chelating iron or by reacting with lipid peroxyl radicals (Doolaege et al., 2012).

Sausage curing might completely or partially denature myoglobin. The supportive effect of globin on Fe^+2^ is disappeared. Ferrous metal (Fe^2+^) ions and free radical as reactive compounds can initiate lipid peroxidation. It is proved that antioxidant components can bind with metal ions such as Fe^2+^ and inhibit free radical formation. As a consequence, the propagation of free radical reactions can be prevented using the chelation of transition metal ions (Aloqbi et al., 2016). In line with our study, Hugo and Hugo (2015) stated that TBA of the samples treated with or/and without PPE enhanced during 20 days of chilled storage. However, Aloqbi et al. (2016) reported that the amount of Fe^2+^‐ferrozine complex significantly reduced in a dose‐dependent manner in the presence of punicalagin in pomegranate juice compared with BHT at the same concentration. In the present study, the rising pattern of PV in SP formulation is ascribed to completely decompose or/and convert α‐ and β‐punicalagin as potential antioxidant to other compounds, showing no peak bands in LC‐mass spectra in the sausages (Figure S1). High antioxidant activity of SE has been identified to be not only based on the main phenolic compounds such as rosmarinic acid, carnosic acid, and carnosol but also some minor constitutes such as monoterpenoids, labdane, sesquiterpenoid, ent‐kaurane, rearranged abietane, abietane, tanshinone, icetexane, clerodane, and pimarane diterpenoids (Asadollahi et al., 2019; Jassbi et al., 2016). The antioxidant properties of carnosol and carnosic acid presumably owing to the presence of a catechol moiety. They act in a hydrogen donator system to interfere with the free radical propagation process (via circumvents oxidation), metal chelators, and singlet O_2_ quenchers which are able to prevent or postpone unsaturated fatty acids and triglycerides against oxidation in bulk and emulsified lipid systems (Birtić et al., 2015; Naveena et al., 2013). Contrary to TBA data, PV of all samples (except SP) represents a reducing pattern that corresponds with the rising trend for TBA values for all sausages. It supposes owing to two reasons: (A) primary oxidation compounds were converted to secondary ones, (B) somewhat decomposition of rosmarinic acid, carnosol, and carnosic acid (in sage) occurred during the storage time at 4°C. Carnosic acid and carnosol were indicated as a scavenger of hydroxyl, DPPH radicals, reactive oxygen species (ROS), or lipid radicals. Mode of action of diterpenes in different in‐vitro systems indicated that the non‐water‐soluble extract (i.e., carnosic acid and carnosol) displayed greater antioxidant capacity and electron donor properties compared to the water‐soluble extract (rosmarinic acid) (Naveena et al., 2013). These findings indicated that higher amount of phenols does not necessarily mean higher antioxidant activity. Both carnosic acid and carnosol can potentially prevent lipid oxidation against reactive oxygen species (ROS)‐induced lipid peroxidation, however, behaving differ route in their reaction with ROS. Carnosol showed resistance to oxidation and its concentration remained constant in the presence of hydroxyl radical or ^1^O_2_ compared to carnosic acid because carnosic acid represents great reactivity toward ROS and is easily oxidizable (Loussouarn et al., 2017). However, loss of carnosol also occurs when subjected to ROS in a lipid medium. This could suppose that carnosol has the potential to interact directly with lipid oxidation‐derived products and can be degraded. Oxidation of carnosic acid was proved by a reduction in the carnosic acid peak and a concomitant production of carnosol (Loussouarn et al., 2017). Consistent with present study, Doolaege et al. (2012) noted that rosmarinic acid, carnosic acid, and carnosol from SE could efficiently retard lipid oxidation in liver pâté. Therefore, it was advisable that sausage samples containing 60 ppm nitrite and SE displayed lower TBA quantities than threshold which can be considered acceptable samples.

#### 3.3.1 Modeling of PV and TBA values of sausage samples

Figure 2d,e show the changes in PV and TBA values as a function of time for six sausage samples. The values of PV in the SP sample increase with time, while in the remaining samples, a decreasing trend for the PV is observed. Figure 2 displays that the amount of TBA in all six sausage samples increases with time.

The data of PV and TBA values were fitted to zero‐, first‐, and second‐order reaction models by plotting *C*, ln (*C*/*C*_*0*_), and 1/*C* against *t*, respectively. The calculated parameters of *k* and *R* ^2^ are presented in Table 2. According to Table 2, the *R* ^2^ values of the second‐order kinetic model have the highest value for most sausage samples, indicating that the changes in PV is better fit to the second‐order model. The results show that the reaction rate constants, *k*, in SN60, SS, and SPN samples are almost equal, and as can be seen, the final values of PV after 14 days are almost the same for all three samples (Figure 2). SSN and SN120 samples have the highest amount of *k*, respectively, after the above three samples. As a result, PV of 14 days for SN120 sample has the lowest value.

On the other hand, in all six sausage samples, the zero‐order reaction model fits the TBA values well. So that the *R* ^*2*^ value in all samples is greater than 0.95. The comparison of the values of the reaction rate constant, *k*, of the samples shows that the SP sample has the highest value of *k*, followed by the SS, SN60, SPN, SN120, and SSN samples, respectively. However, compared to SP, the values of *k* of these five samples are very close to each other. In general, SP and SSN samples have the highest and lowest 14‐day TBA value, respectively.

### 3.4 Color evaluation

#### 3.4.1 L*, a*, and b* values

Color measurement of meat products and their appearance is considered as essential part of meat researches, implying freshness and wholesomeness, which, in turn, the customer's decisions to accept or reject foodstuff. Thus, the inhibition of detrimental changes in meat pigment is crucial (Ghaderi‐Ghahfarokhi et al., 2016). L*, a*, and b* of treated sausages with sage and pomegranate during chilled storage are exhibited in Table 4.

**TABLE 4:** L*, a*, b*, oxymyoglobin (%), and metmyoglobin (%) of sausage treated with pomegranate and salvia extract after first, 7th, and 14th day of storage.

Sausage samples	Storage time																				
	First			7			14			First		7		14		First	7	14	First	7	14
	L*	a*	b*	L*	a*	b*	L*	a*	b*	Oxy (%)	Met (%)	Oxy (%)	Met (%)	Oxy (%)	Met (%)	Nitrite residue (mg/kg)			N‐nitrosodimethylamine (μg/kg)		
SN120	68.3 ± 0.5aA	22.2 ± 1.1 aA	10.5 ± 1.4 bD	61.4 ± 2.7 bA	18.7 ± 1.1bA	10.0 ± 1.0bE	58.2 ± 1.7 cA	16.0 ± 1.4 cA	11.6 ± 0.8 aE	84.8 ± 3.8aA	22.6 ± 1.8cC	76.2 ± 2.5bA	28.7 ± 1.1bD	65.4 ± 1.7cA	31.0 ± 2.4aE	101.54 ± 2.42aA	76.98 ± 4.61aB	68.09 ± 3.38aC	ND	4.82 ± 0.36aB	5.43 ± 0.27aA
SN60	62.6 ± 1.5 aB	16.0 ± 0.1 aB	12.3 ± 0.4cC	59.1 ± 1.2 bB	13.2 ± 0.4 bB	14.3 ± 0.5 bC	58.0 ± 0.7bA	12.6 ± 2.5 bB	15.8 ± 1.3aC	76.8 ± 2.7aB	28.4 ± 1.5bB	70.2 ± 2.5bB	32.3 ± 2.1aC	58.4 ± 1.7cB	33.9 ± 1.4aD	88.49 ± 2.42bA	52.31 ± 3.25bB	38.19 ± 3.55bC	ND	1.82 ± 0.13bB	2.38 ± 0.25bA
SS	62.0 ± 2.1 aB	5.0 ± 0.5 aD	14.0 ± 0.6cB	56.0 ± 1.0 bC	4.1 ± 0.3 bE	15.8 ± 0.4 bB	51.3 ± 1.1 cC	4.0 ± 0.3 bE	17.3 ± 1.0aB	39.8 ± 2.3aE	34.8 ± 1.5cA	33.7 ± 1.7beE	38.7 ± 1.9bB	31.8 ± 1.5cD	53.2 ± 2.4aB	ND	ND	ND	ND	ND	ND
SSN	66.6 ± 2.1 aA	15.3 ± 0.8 aB	12.0 ± 1.2bC	59.3 ± 1.8 bB	12.0 ± 0.7 bC	13.0 ± 0.5aD	58.5 ± 0.5 bA	10.2 ± 1.5 cC	13.2 ± 0.6 aD	53.2 ± 2.6aC	23.8 ± 1.2aC	49.9 ± 2.1bC	31.7 ± 1.1bC	39.3 ± 1.9cC	32.7 ± 1.5bD	49.56 ± 1.24dA	23.28 ± 2.05cB	13.14 ± 1.15cC	ND	0.42 ± 0.07cB	0.74 ± 0.05cA
SP	52.0 ± 2.4 aD	3.6 ± 0.5aE	15.2 ± 0.7 cA	47.0 ± 1.3bD	2.0 ± 0.2 bF	16.9 ± 1.1 bA	46.2 ± 1.3 bD	1.6 ± 0.6 bF	23.2 ± 1.0aA	38.5 ± 2.4aE	29.3 ± 1.8cB	30.5 ± 2.8bF	46.3 ± 1.8bA	24.6 ± 1.5cE	63.2 ± 2.4 aA	ND	ND	ND	ND	ND	ND
SPN	57.9 ± 1.8 aC	9.2 ± 0.5 aC	14.0 ± 0.3bB	55.3 ± 1.8 bC	8.1 ± 1.2 bD	17.0 ± 0.6aA	54.1 ± 2.4 bB	7.6 ± 0.5 bD	17.5 ± 0.5aB	47.6 ± 2.3dD	23.3 ± 1.8 cD	43.8 ± 1.5 bD	29.7 ± 1.8bD	38.7 ± 2.8cC	37.2 ± 2.0 aC	83.87 ± 3.50cA	48.14 ± 4.19bB	35.26 ± 2.42bC	ND	1.77 ± 0.24bB	2.12 ± 0.12bA

*Note*: Values are the average of triplicates ± standard deviation. Different lowercase letters in each column and capital letters in each row indicate significant statistical difference (*p* ≤ .05). Same letters in each column and row indicate no significant statistical difference (*p* ≥ .05). SN120 (sausage containing nitrite 120 ppm), SN60 (sausage containing nitrite 60 ppm), SS (sausage containing SE extract 1%), SP (sausage containing PEP extract 1%), SPN (sausage containing PEP extract 1% + nitrite 60 ppm), SSN (sausage containing SE extract 1% + nitrite 60 ppm).

L* values of sausages illustrated decreasing trend during storage. As can be seen, L* value of SN120 (58.2 ± 1.7), SN60 (58.0 ± 0.7), and SSN (58.5 ± 0.5) were obviously more than others, after 14‐day storage time. The lowest L* value of sausage was measured in SP (46.2 ± 1.3). Brown color development occurs by Maillard reaction (He et al., 2012). The lipid oxidation process results in increased myoglobin oxidation (metmyoglobin) and meat discoloration. Regarding the results obtained by Gardeli et al. (2019), changes in color of sausage containing pomegranate can be induced by anthocyanins decomposition as well as the interaction between anthocyanins and condensed tannins that could favor the formation of polymeric anthocyanins. He et al. (2012) and Naveena et al. (2013) reported that the addition of pomegranate juice and rind pomegranate powder extract changed the chicken meat patties from pale raw to grayish color (He et al., 2012; Naveena et al., 2013).

In the case of a* values, the surface color of sausages was influenced by formulation at any storage time (*p* < .05). A substantial reduction of a* value in differently treated samples was found through the storage period (*p* < .05) (Table 4). This was associated with the conversion of red oxymyoglobin to brown metmyoglobin. The reducing a* parameter is in line with results obtained by Hugo and Hugo (2015) in pomegranate‐treated raw pork upon 9 days of refrigerated storage time and Hugo and Hugo (2015) in rosemary extracts‐treated pork meat.

The increased rate pattern observed for b* values during refrigerated storage time was as follows; SN120 (10.5–11.6) ˂ SSN (12.0–13.2) ˂ SN60 (12.3–15.8) ˂ SPN (14–17.5) and SS (14–17.3) ˂ SP (15.2–23.5) (Table 4). Although b* values of the samples treated with PPE and SE rapidly faded (*p* < .05), yellowness decreasing rate was displayed in those treated with combination of SE and nitrite during storage time. The increase in b* parameter may be ascribed to facilitate polyphenol degradation, lipid oxidation, and formation of metmyoglobin. Similarly, Hugo and Hugo (2015) stated increased b* values in pomegranate and rosemary‐treated raw meat.

#### 3.4.2 Oxymyoglobin (%) and metmyoglobin (%) content

The desirable red color of the meat is commonly related to the ratio proportion of red myoglobin, bright‐red oxymyoglobin, and gray‐brown metmyoglobin (Ghaderi‐Ghahfarokhi et al., 2016). According to consumer's preference, the percent of MetMb should not exceed greater than range 30%–40% of total pigments on the surface of fresh meat. OxyMb of all sausages decreased when the storage time increased (Table 4) which was accompanied by the increase in MetMb content. The order of OxyMb content of sausage formula was as follows SN120 > SN60 > SSN > SPN > SS > SP. Meanwhile, the decline of OxyMb level was more considerable within the first 7 days of storage for all treated samples. OxyMb content of SN120 and SN60 was significantly (*p* ˂ .05) greater than other formulations. The reduction of ferric‐metmyoglobin (Fe^3+^) to ferrous‐oxymyoglobin (Fe^2+^) simplifies by adding nitrites. The pomegranate extract represented lowest effect on protection of OxyMb because of the degradation of punicalagin α and β while the bioactive compounds of sage (rosmarinic acid, carnosic acid, and carnosol) cause to protect of OxyMb. Therefore, higher protection of OxyMb was exhibited in sausages incorporated with sage extract and nitrite compared with sausage containing sage extract and this impact was conspicuous from 7 days of storage time onwards. Besides, a significant negative correlation was observed between OxyMb pigment and lipid oxidation. In the present study, TBA data were in line greatly with OxyMb content (*r* ^2^ = −.76) and a* (*r* ^2^ = −.85), implying a reduction in OxyMb content and a* with enhancement in TBA value during refrigerated storage time. As a result, SN120, SN60, and SSN can be assumed to have more OxyMb content (>40%) at the final period of storage. Similarly, thyme essential oils + ascorbic acid (Ghaderi‐Ghahfarokhi et al., 2016) could postpone reduction of OxyMb in minced beef burgers during cold storage. In contrast, Michalczyk et al. (2012) stated neither addition of essential oils of hyssop and coriander nor the storage temperature substantial influence the amount of OxyMb of ground beef following 15‐day storage at 6°C. However, the gradual enhancement in the amount of MetMb during storage was accompanied by the increase in the TBA in sausage formulations. A negative relation between a* and MetMb content was also reported for raw pork burgers incorporated with *Moringa oleifera* leaf extract during storage at 4°C (Falowo et al., 2017; Muthukumar et al., 2014; Shah et al., 2015). Liu et al. (2015) stated that beef patties treated with several natural antioxidants led to increase in the content of MetMb after 6 days of storage and then reduce MetMb concentration after 8 days of storage.

### 3.5 Biogenic amine accumulation

**FIGURE 3:**
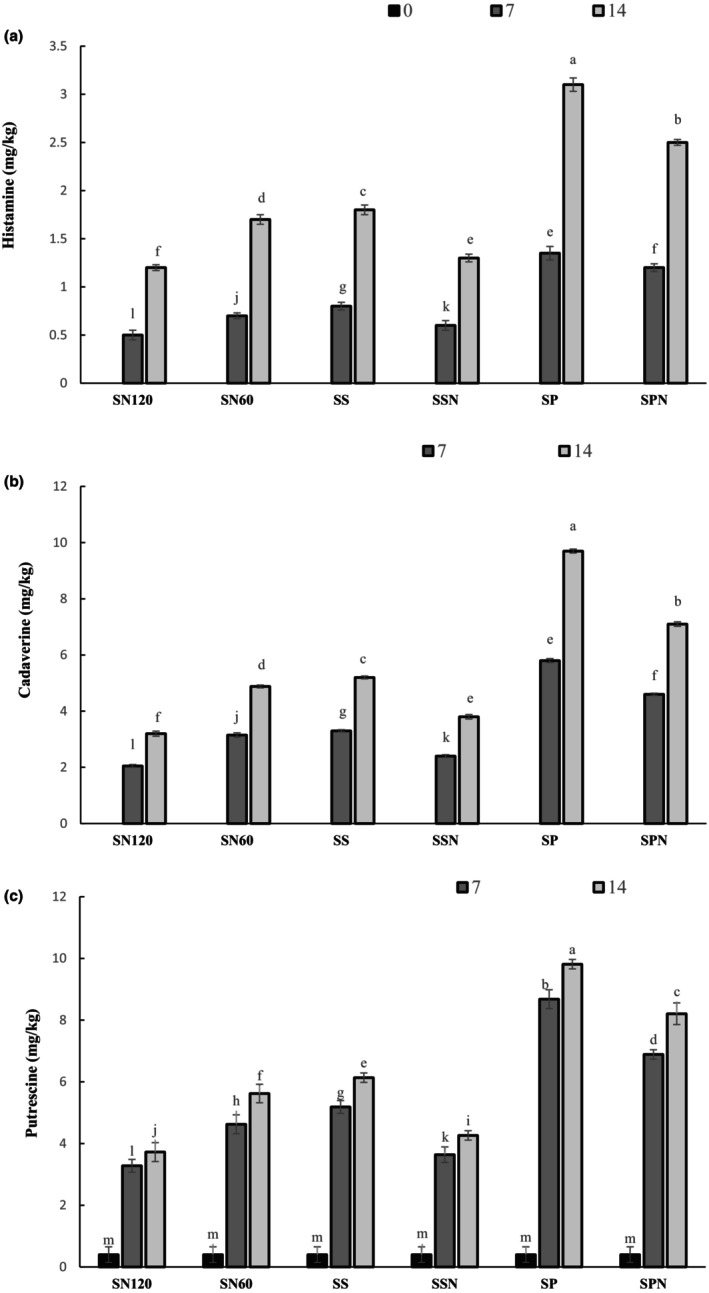
Changes in the amount of cadaverine (a), histamine (b), and putrescine (c) in sausage treated with SE and PPE during storage time (0: black, 7th day: dark gray, 14th day light gray).

Biogenic amines are organic bases with aliphatic, aromatic, or heterocyclic structures that are mainly produced from decarboxylation of amino acid by microbes in food, especially in meat and fish products. As shown in Figure 3a–c, putrescine is the most abundant biogenic amine in this study, followed by cadaverine and histamine. Raw material was found to be hygienic since cadaverine and histamine were not detected. During ripening, putrescine content (mg/kg) increased gradually with different rates depending on sausage formulation, the amount of nitrite, and extract type after 14 days of storage (*p* < .05) in the SN120 (3.9), SN60 (5.7), SS (6.1), SSN (4.1), SP (10.2), and SPN (8.2). The same trend was observed for the amount of cadaverine and histamine as SN120 (3.4 and 1.2), SN60 (4.9 and 1.7), SS (5.3 and 1.8), SSN (3.7 and 1.4), SP (8.8 and 3.1), SPN (7.1 and 2.5) following 14‐day storage time. SSN (combination of sage extract and 60 ppm nitrite) could progressively inhibit the formation of biogenic amines (putrescine, histamine, and cadaverine). On account of total degradation of α‐ and β‐punicalagins of PPE, SP could not postpone the formation of biogenic amines. In several cases, cadaverine is caused by the activity of decarboxylase‐positive bacteria, such as Enterobacteria (*E. coli*) that are not completely inhibited by curing. The amount of *E. coli* in sausage containing SE was significantly lower than PPE, causing reduction in decarboxylase activity and also resulting in cadaverine. Like cadaverine, the highest amount of putrescine was observed in sausage treated with PPE, while the lowest was in the SN120 and SSN at the end of 2‐week storage. Similarly, it is noteworthy that combined SE and nitrite (60 ppm) displayed the strongest impact on inhibiting the generation of histamine. Unlike putrescine and cadaverine, the change in histamine levels did not show variation during storage. The main reason behind this is the production of histamine depends greatly on histidine decarboxylase, an enzyme whose activity is suppressed at low storage temperatures (4°C) compared to its optimum temperature (30°C) (Roseiro et al., 2010). Consistent with our study, Wang et al. (2015) reported that α‐tocopherol, green tea extract, grape seed extract, and their combinations reduced putrescine production of dry‐cured bacon compared to the control sausage.

### 3.6 Residual nitrites and NDMA content

As seen in Table 4, the residual nitrite contents in the sausages treated with SE reduced more quickly than those with PPE and reached to 13.14 and 35.26 mg/kg at the end of ripening of sausage containing SE and PPE. It is demonstrated that numerous factors, mainly raw meat type, initial nitrite level, meat pH, storage temperatures, and the presence of reductants can affect nitrite depletion. When the meat pH reduces to less than 6.0, the nitrite can be turned into nitrous acid or nitric oxide, which can react with polyphenols or endogenous substances, reducing nitrite residues (Wang et al., 2015). Such reductions in residual nitrite were probably due to the reaction of the bio‐compounds present in plant polyphenols. As found in Table 2, the reduction pattern in the amount of rosmarinic acid, carnosol, and carnosic acid compounds in sage extract was lowered from 313.13–258.06, 41.61–36.08, and 86.94–82.46 mg/kg, before adding to sausage formulation, respectively, to 297.53–216.44, 40.62–33.15, and 81.78–76.73 mg/kg in SSN sausage further 14th day of storage time. It could be implied that SE had nitrite scavenging ability due to bioactive compounds such as rosmarinic acid, carnosol, and carnosic acid.

However, the use of the PPE (SPN) had no statistical influence on the residual nitrite with SN60 owing to total decomposition of α‐ and β‐punicalagin in PPE during storage. Meanwhile, nitrogen compounds in muscle matrix are dynamically transformed during storage, which may explain the gradual decrease in residual nitrite content regardless of treatment (De Mey et al., 2014). A similar trend was also reported by Li et al. (2013) and Wang et al. (2015) for dry‐cured sausages, indicating that green tea and grape seed polyphenols substantially declined residual nitrite. In line with the present study, Deng et al. (2022) incorporated tea polyphenol, apple polyphenol, and cinnamon polyphenol into dry‐fried bacon and concluded that plant polyphenols potentially can be used as natural antioxidants for reducing nitrite application level while also ameliorating the safety of bacon.

The amount of NDMA gradually augmented during the storage regardless of treatment (Table 4). The highest NDMA content was observed in the SN120 and the lowest in those treated with SSN throughout the whole processing stages. After storage for 2 weeks, NDMA concentration reached 2.12 and 0.74 mg/kg in SPN and SSN, respectively. The changes in NDMA levels over storage might depend on chemical reactions between NDMA precursors (residual nitrite) in meat products (Wang et al., 2015).

### 3.7 Antimicrobial activity

**FIGURE 4:**
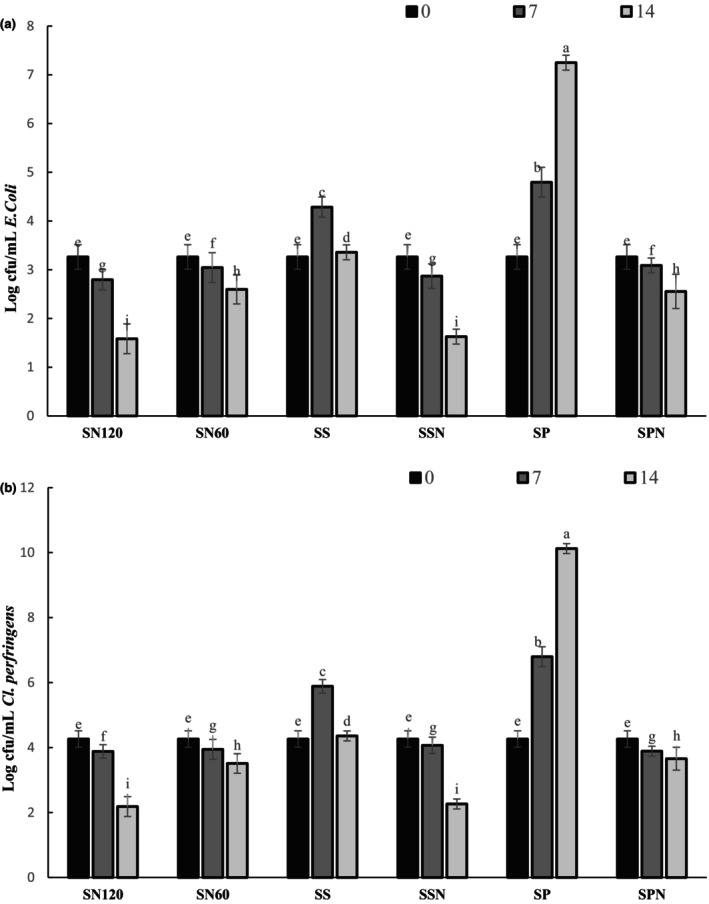
Changes in the count of *E. coli* (a), and *Cl. perfringens* (b) in sausage treated with SE and PPE during storage time (0: black, 7th day: dark gray, 14th day light gray).

The antimicrobial activity of PPE and SE incorporated into sausage in the absence and presence of nitrite against common food spoilage and pathogenic bacteria including *Cl. perfringens* and *E. coli* were investigated. *E. coli* and *Cl. perfringens* were around 4.4 and 3.2 log cfu/g in raw material. After 2 weeks of storage, *E. coli* and *Cl. perfringens* counts in the SN120 and SSN were significantly lower than the other samples (*p* < .05), with the values 2.1 and 1.5 log cfu/g for SN120 and 2.2 and 1.6 log cfu/g for SSN formulation (Figure 4). Our results discovered that adding antioxidants for sausage curing can effectively reduce *E. coli* and *Cl. perfringens* populations during ripening and storage. Different studies revealed that most plant extracts are inefficient against gram‐negative organisms. Compared to previous results gained with diverse aqueous extracts, the obtained data are similar in some cases and even better (Kanatt et al., 2010; Yin et al., 2016). The most probable mechanism of antimicrobial activity by phenolic compounds has been assumed to be owing to the disruption of the cell membrane and increased membrane permeability (Ibrahim et al., 2011).

## 4 CONCLUSIONS

Agro‐industrial by‐products including pomegranate peel or sage leaves are leading sources of phenolic compounds that possess high antimicrobial and antioxidant properties. Although nitrite exerts many positive influences as a curing agent, lower residual nitrite contents in meat products require to minimize the risk of nitrosamine generation as carcinogenic compounds. The addition of SE to sausage formulation could progressively postpone lipid oxidation and discoloration. Moreover, could reduce residual nitrites and NDMA content during refrigerated storage due to residual rosmarinic acid, carnosol, and carnosic acid in extract‐treated sausage. However, oxidation indices, residual nitrites, NDMA, and biogenic amine increased in sausage samples containing PPE extracts on account of total degradation of α‐ and β‐punicalagin during storage.

The changes in rosmarinic acid and carnosic acid with time were evaluated by the first‐order kinetic model. According to the *k* values, these compounds are degraded at a higher rate in SSN sample than in SS sample. Moreover, PV changes with time were in good agreement with the second‐order kinetic model, while the zero‐order reaction model had the best fit with the TBA values. Furthermore, it was manifested that the addition of sodium nitrite dose to sausage formulation could be declined from 120 to 60 mg/kg when SE (1%) was utilized.

## AUTHOR CONTRIBUTIONS

**Elahe Abedi:** Conceptualization (equal); data curation (equal); validation (equal); writing – original draft (equal); writing – review and editing (equal). **Atefeh Tavakoli:** Formal analysis (equal); project administration (equal). **Simin Zamanizadeh:** Investigation (equal); methodology (equal). **Shahrzad Maleki:** Data curation (equal); software (equal). **Amir Reza Jassbi:** Funding acquisition (equal); investigation (equal); visualization (equal); writing – original draft (equal); writing – review and editing (equal).

## FUNDING INFORMATION

This work was supported by medicinal and natural products chemistry research center, Shiraz University of Medical Sciences.

## CONFLICT OF INTEREST STATEMENT

The authors declare that they have no conflict of interest.

## ETHICS STATEMENT

This article does not contain any studies with human participants or animals performed by any of the authors.

## Supporting information


Figure S1.

## Data Availability

Research data are not shared.
