# Supplementary material for: The correlation among residual nitrites, biogenic amines, N‐nitrosamine formation, and degradation occurrence of punicalagin α/β, rosmarinic acid, carnosol, and carnosic acid in extract‐treated sausage during storage
Source: Food Sci Nutr. 2023 Jun 15;11(9):5409–26. doi: 10.1002/fsn3.3498 (PMC10494630; doi:10.1002/fsn3.3498)
Supplement: Supplementary file 1 — Figure S1. [file FSN3-11-5409-s001.docx]

1. **B)**

**C)**

**D) E)**

**F)**

**Fig. 1S.** Chromatogram of pomegranate with methanol before addition to sausage (A), sausage sample containing pomegranate extract at first day (B), sausage sample containing pomegranate extract at 14^th^ day (C). Salvia *eremophila* crude extract with methanol (D), sausage sample containing salvia extract at first day (B), sausage sample containing salvia extract at 14^th^ day (C).
